# Unveiling the intriguing relationship: oncogenic *KRAS* , morphological shifts, and mutational complexity in pancreatic mucinous cystic neoplasms

**DOI:** 10.1002/path.6397

**Published:** 2025-02-05

**Authors:** Lucas‐Alexander Schulte, Annika Beck, Ralf Marienfeld, Ninel Azoitei, Thomas FE Barth, Alica Beutel, Vladimir Benes, Markus W Büchler, Nadine Therese Gaisa, Katja Kilani, Nathalia Giese, Christoph W Michalski, Peter Möller, Lukas Perkhofer, Tobias Rausch, Stefan Repky, Elodie Roger, Jeanette Scheible, Thomas Seufferlein, Peter Schirmacher, Andreas Wolfgang Berger, Thilo Hackert, Alexander Kleger

**Affiliations:** ^1^ Division of Interdisciplinary Pancreatology, Department of Internal Medicine 1 Ulm University Hospital Ulm Germany; ^2^ Department of Pathology Ulm University Hospital Ulm Germany; ^3^ Institute of Molecular Oncology and Stem Cell Biology (IMOS) Ulm University Hospital Ulm Germany; ^4^ Department of Internal Medicine 1 Ulm University Hospital Ulm Germany; ^5^ Genomics Core Facility European Molecular Biology Laboratory (EMBL) Heidelberg Germany; ^6^ Department of General, Visceral and Transplantation Surgery University of Heidelberg Heidelberg Germany; ^7^ EPZ‐Biobank, Division of Pancreatic Cancer Research, Department of General, Visceral and Transplantation Surgery University of Heidelberg Heidelberg Germany; ^8^ Institute of Epidemiology and Medical Biometry Ulm University Ulm Germany; ^9^ Institute of Pathology University Hospital Heidelberg Heidelberg Germany; ^10^ Evangelisches Krankenhaus Königin Elisabeth Herzberge Department of Gastroenterology Berlin Germany; ^11^ Department of General Surgery University Hospital Hamburg Eppendorf Hamburg Germany

**Keywords:** pancreatic ductal adenocarcinoma (PDAC), mucinous cystic neoplasms (MCN), KRAS mutations, intratumor heterogeneity, variant allele frequency (VAF), circulating tumor DNA (ctDNA), tumor progression, mutational landscape

## Abstract

Pancreatic ductal adenocarcinoma (PDAC) often arises from preexisting cystic lesions such as intraductal papillary mucinous neoplasms (IPMN) and mucinous cystic neoplasms (MCN). This study investigated the molecular heterogeneity and mutational landscape of MCN in relation to PDAC, highlighting the significance of *KRAS* mutations in tumor progression. Utilizing targeted next‐generation sequencing on low‐grade MCN and invasive PDAC samples, we identified a substantial overlap in mutational profiles, particularly mutations in *KRAS*, *TP53*, and *FBXW7*. Specifically, 69.2% of MCN exhibited somatic mutations, with *KRAS* mutations being a predominant oncogenic driver. The characterization of mutant versus wildtype *KRAS* variant allele frequencies (VAF) indicated higher mutation levels in PDAC compared to MCN, suggesting an evolutionary trajectory toward malignancy. Further histological analysis of 12 additional MCN cases revealed significant intratumor heterogeneity, with variant *KRAS* mutation distributions correlating with distinct cellular morphologies and dysplastic features. Additionally, we explored the potential of liquid biopsies, demonstrating a concordance rate of 71.4% for *KRAS* mutation detection in circulating tumor DNA (ctDNA) relative to tissue biopsies across cohorts. Our findings underscore the relevance of evaluating *KRAS* mutations—herein referred to as VAF per microdissected region—as they relate to histopathological markers of dysplasia, contributing to improved stratification of pancreatic lesions and facilitating personalized treatment strategies. In conclusion, this comprehensive analysis of MCN highlights the importance of *KRAS* as a crucial biomarker for both malignant progression and therapeutic decision‐making in pancreatic pathology. Ultimately, our study suggests that characterizing the mutational landscape and histological features of MCN can enhance early detection and intervention strategies for at‐risk patients. © 2025 The Author(s). *The Journal of Pathology* published by John Wiley & Sons Ltd on behalf of The Pathological Society of Great Britain and Ireland.

## Introduction

A relevant proportion of human pancreatic ductal adenocarcinoma (PDAC) arises from cystic neoplasms such as intraductal papillary mucinous neoplasms (IPMN) and mucinous cystic neoplasms (MCN). The incidental identification of these lesions during abdominal imaging offers the opportunity for preventive strategies in patients called ‘at‐risk’ for PDAC [1, 2, 3] and tailored therapy to justify or to circumvent pancreatic resection that is associated with significant morbidity and mortality [2, 4, 5]. Understanding the precise molecular alterations paves the way to discover clinically useful biomarkers to track malignant progression and to implement personalized therapy. MCN are large mucus‐filled cysts lined by cuboidal mucinous epithelium surrounded by an ovarian‐like stroma. While this epithelium can exhibit various morphological atypia up to frank carcinoma, its mutational landscape and, even further, its underlying heterogeneity remains largely unknown [6].

## Materials and methods

### Case selection and histopathological assessment

MCN cases were retrospectively identified, and MCN formalin‐fixed, paraffin‐embedded (FFPE) tissue for panel sequencing, as well as plasma samples, were obtained from the biorepository of the University Hospital of Heidelberg, Germany. Additional samples of an equally sized group of pancreatic carcinoma specimens were also obtained from the same biorepository. Inclusion criteria for MCN‐1 Cohort and MCN‐3 Cohort were as follows: (i) age 18 years or older; (ii) no prior therapy; (iii) histopathological‐confirmed MCN; (iv) exclusion of invasive cancer. All PDAC cases had to be nonmetastatic. The MCN‐1 and PDAC cohorts were matched for age. For MCN‐2 Cohort, the pathology database at the University Hospital Ulm, was reviewed for cases diagnosed with mucinous cystic neoplasm. Each identified case was re‐evaluated by a trained pathologist to confirm the MCN diagnosis. If the diagnosis was confirmed and sufficient remaining FFPE tumor tissue was available for analysis, the case was included. MCN FFPE tissue for assessment of intratumor heterogeneity was retrospectively identified and obtained from the biorepository of the University Hospital Ulm and Heidelberg, Germany. Local ethics approval was given (No S‐708/2019, 43/2020, and EVE/2012/2.0, respectively). Slides were reassessed to confirm the correct diagnosis and for evaluation of dysplasia by an experienced pathologist. Criteria for dysplasia were used according to the current WHO classification [7].

### MCN characterization

Dichotomous criteria used for evaluation were cell shape (flat, cuboidal, or columnar), papillary growth, pseudo‐stratification of nuclei, loss of polarity, nuclear atypia, complexity of architecture, mitotic count, necrosis, and invasion. After excluding areas that could not unequivocally be graded or appeared different on the microdissection slides, as well as normal parenchyma and cancerous areas, 114 regions remained and were statistically compared.

### Library preparation and next‐generation sequencing

For library preparation, the NEBNext DNA Ultra II Kit (New England Biolabs, Frankfurt Am Main, Germany) was used with 10 polymerase chain reaction (PCR) cycles for all FFPE DNA samples. Since the FFPE fixation and storage damages nucleic acids, we used solid‐phase reversible immobilization (SPRI) beads to remove short DNA fragments before processing. Final libraries were sequenced on an Illumina HiSeq‐4000 sequencing instrument (Illumina, San Diego, CA, USA) in paired‐end mode (2×75 bp) at the EMBL GeneCore.

### Whole‐genome alignment and copy‐number variant calling

Sequenced fragments were aligned to the human reference genome (hg19) using bwa [8]. Alignments were sorted and indexed using samtools [9] and quality controlled using Alfred [10]. FreeBayes was employed to call and genotype short variants [11], which were subsequently normalized and left‐aligned using bcftools [10]. Delly was used to call structural and copy‐number variants [12]. The log2 normalized read‐depth signal of the tumor genome and matched control was segmented using the DNAcopy Bioconductor package [13]. Using DNAcopy, we did not detect any large (>2 Mbp) clonal somatic rearrangements in high‐confidence regions of the genome in the tumor samples.

### Tissue microdissection

Target regions for microdissection were identified on standard hematoxylin–eosin (HE)‐stained sections. Afterwards, serial sections were sampled using a laser microdissection microscope (Zeiss PALM Micro Beam, Oberkochen, Germany). For panel sequencing, the entire neoplastic epithelium of MCN and representative areas of adjacent normal pancreatic parenchyma or pancreatic ductal adenocarcinoma (PDAC) carcinoma specimens were collected. To minimize stromal contamination, the cutting edge of the laser‐microscope was positioned as close to the basement membrane as possible, while preserving nuclear integrity of the neoplastic epithelium. The dissection process was monitored in real time by a board‐certified pathologist. For the assessment of intratumoral heterogeneity, target regions were chosen according to morphological heterogeneity in terms of growth pattern, and cellular and nuclear architecture. If possible, different regions were chosen from the same cyst. DNA was extracted according to standard procedures (Qiagen® DNA extraction kits, Hilden, Germany).

### Genetic assessment

For panel sequencing, a custom panel including the nine most commonly mutated genes in pancreatic carcinoma was created and sequencing was performed according to standard procedures on an Illumina® sequencing platform. Genes included were *KRAS, TP53, RNF43, FBXW7, APC, SMAD4, PIK3CA, CDKN2A*, and *ATM*. For droplet digital PCR, a multiplex assay covering *KRAS* mutations G12A, G12C, G12D, G12R, G12S, G12V, and G13D (Bio‐Rad®, Hercules, CA, USA) was used following the manufacturer's manual. Samples were considered positive when the Poisson probability of false positivity was below 0.05.

### Statistical analysis and visualization

For statistical analyses, R Studio (R v. 4.2.2; Boston, MA, USA) was used. For visualization, R and GraphPad Prism™ version 9 (Graphpad Inc., Boston, MA, USA) were used.

## Results

**Figure 1:**
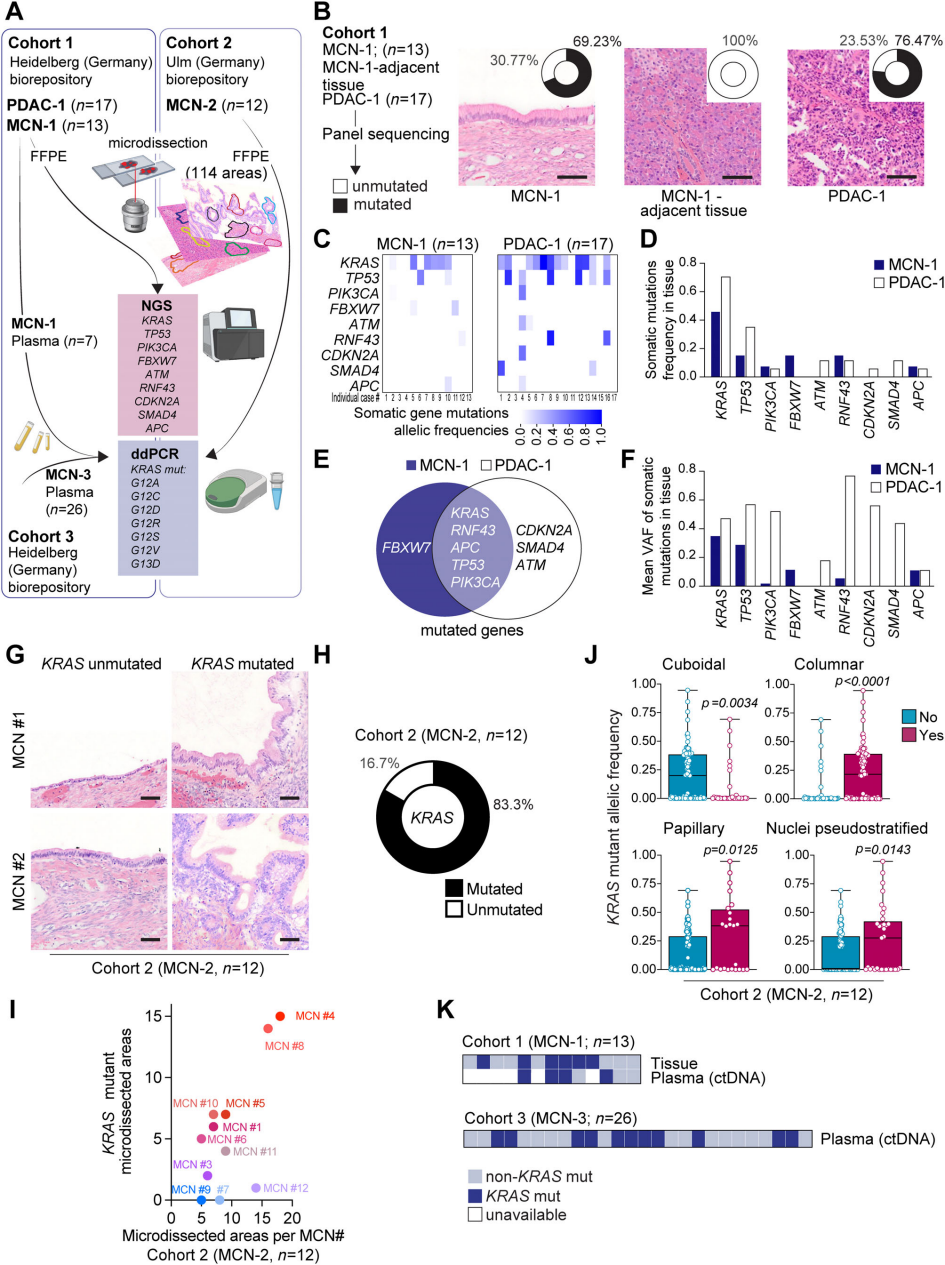
Comprehensive analysis of *KRAS* mutations and histological features in MCN. (A) Schematic representation of the workflow used to investigate the mutational status of PDAC and MCN samples and introduction of the three independent cohorts (Cohort 1, MCN‐1, and PDAC‐1, Heidelberg: tissue and partially plasma; Cohort 2, MCN‐2, Ulm: only tissue; Cohort 3, MCN‐3, Heidelberg: only plasma). (B) Left: Scheme of the chosen panel sequencing approach to analyze Cohort 1 (tissue of PDAC and MCN). Right: Histologic sections of PDAC, MCN, and MCN adjacent tissues from Cohort 1 stained by hematoxylin–eosin (HE). The circular diagrams inlaying each image display the mutational burden across the three types of analyzed tissue. (C) Heatmap showing somatic gene mutations allelic frequencies in the MCN‐1 (left panel) and PDAC‐1 (right panel) cases from Cohort 1 (D) Somatic mutations frequency in tissue from the MCN‐1 and PDAC‐1 Cohort 1. (E) Venn diagram showing the overlap of mutated genes between MCN‐1 and PDAC‐1 cases from Cohort 1. (F) Mean of variant allelic frequencies of somatic mutations in tissue from the MCN‐1 and PDAC‐1 Cohort 1. (G) Histologic sections of unmutated and mutated *KRAS* MCN from Cohort 2. (H) Circular diagram indicating the percentage of MCN cases in Cohort 2 carrying at least one microdissected area per MCN a *KRAS* G12/G13 mutation as measured using ddPCR. (I) Diagram showing total amount of microdissected areas and corresponding number of *KRAS* mutant areas per MCN case in Cohort 2. (J) *KRAS* mutant allelic frequency in cuboidal, columnar, papillary, and nuclei pseudostratified regions of MCN from Cohort 2. (K) Heatmap depicting the *KRAS* mutational status in the tissue and in a corresponding plasma ctDNA sample, if available, from the MCN cases in Cohort 1 (upper panel). *KRAS* mutational status assessed in Cohort 3 for which only plasma samples but no tissues were available (lower panel). Panel A was created with BioRender.com.

The present study aimed to characterize the molecular heterogeneity across various MCN cohorts (supplementary material, Tables S1 and S2) with distinct methods and approaches (schematic in Figure 1A). First, a cohort of 13 low‐grade MCN and 17 age‐matched, invasive, untreated, and non‐MCN‐derived PDAC (Figure 1B; Cohort 1; epithelium MCN‐1; adjacent tissue‐MCN‐1; PDAC‐1) was subjected to next‐generation panel sequencing of genes commonly mutated in MCN [14] (*KRAS, TP53, RNF43, FBXW7, APC, SMAD4, PIK3CA, CDKN2A, ATM*). The entire MCN epithelium from several consecutive sections was microdissected and pooled before DNA extraction (Figure 1A). The target coverage was 3,803. In nine out of 13 MCN epithelia (69.2%) and 13 out of 17 PDAC cases (76.47%), we detected at least one somatic class 4/5 mutation (MCN: range 1–3; PDAC: 1–5). MCN‐adjacent healthy tissue did not comprise any mutations (Figure 1B–D; not shown). The most frequently mutated genes in MCN were *KRAS* (*n* = 6; 46.2%), *TP53*, and *FBXW7* (*n* = 2 each; 15.3%) followed by *PI3KCA, RNF43*, and *APC* (*n* = 1 each; 7.7%) (Figure 1C,D). Similarly, *KRAS* and *TP53* mutations were top‐ranked in PDAC, with *FBXW7* being the sole gene not found mutated in any of the sequenced PDACs and *CDKN2A, SMAD4*, and *ATM* not mutated in any of the sequenced MCN (Figure 1D,E). Variant allele frequencies (VAF) of the mutations were generally higher in PDAC than MCN (Figure 1F). A subset of MCN was applied also to low coverage whole‐genome sequencing (WGS); however, we neither detected copy number variants (CNV) nor reidentified any of the mutations in line with the generally low MCN VAF (supplementary material, Figure S1A–D). Overall, this analysis highlights a relevant overlap between low‐grade MCN and invasive PDAC mutational landscapes, identifying *KRAS* as a key oncogenic driver of MCN, with VAF best segregating the two entities.

High levels of mutant *KRAS* are linked to aggressive PDAC phenotypes and specific copy number aberrations, impacting clinical outcomes. Recent studies indicate that measuring *KRAS* dosage can provide insights into tumor aggressiveness, guide treatment decisions, and improve personalized medicine, while emphasizing the need for standardized methods and integration with other biomarkers in clinical trials [15, 16, 17, 18, 19]. Its impact in rare MCN remains unexplored. To correlate the presence of oncogenic *KRAS* with histomorphologic features and intratumor heterogeneity, we microdissected 114 distinct areas across 12 additional MCN cases (Figure 1A and supplementary material, Figures S2–S13; Cohort 2, MCN‐2). Notably, each MCN showed substantial heterogeneity in (i) cellular shape (cuboidal, columnar, flat) in 12 cases; (ii) nuclear localization and shape with focal differences in basal nuclei (*n* = 9), nuclear atypia (*n* = 8), and pseudostratified nuclei (*n* = 8); and (iii) regional papillary growth (*n* = 6). Loss of polarity and higher mitotic count were documented in individual regions (supplementary material, Table S2 and Figure 1G). Ten of these 12 MCN (83.3%) had detectable *KRAS* G12/G13 mutations as measured by digital droplet PCR (ddPCR; Figure 1H). *KRAS* mutations were heterogeneously distributed across the different regions analyzed per MCN: one MCN carried a *KRAS* mutation with low VAF (0.013) in only 1 of 14 regions; three were entirely mutated (mean: 6.7 regions, mean VAF = 0.31); two were all‐negative (mean: 6.5 regions), and in seven cases regions with exclusively wildtype *KRAS* and mutant *KRAS* alternated (mean: 12.2 regions, mean VAF *KRAS* mutant areas 0.19) (Figure 1I and supplementary material, Table S2). Overall, we found a significant association between mutant *KRAS* allelic frequency and cell shape (columnar, *p* < 0.0001, versus cuboidal, *p* = 0.0034), papillary growth pattern (*p* = 0.0125), and pseudostratified nuclei (*p* = 0.0143), linking *KRAS* mutation‐presence or dosage to dysplasia in MCN (Figure 1J). However, the overall tumor grade did not correlate with mutant *KRAS* allelic frequency.

To probe this observation as a putative biomarker from a liquid biopsy perspective, as previously reported for IPMN [1], the concordance of *KRAS* mutations in circulating tumor DNA (ctDNA) and tumor tissue DNA was assessed by ddPCR in seven MCN with an available plasma specimen from Cohort 1 (Figure 1A). The *KRAS* G12/G13 mutation rate was 57% in the blood specimens, leading to an overall concordance rate between blood and tissue of 71.4% (Figure 1K). Specifically, in three out of seven MCN cases, detectable *KRAS* mutations were reported in both tissue and ctDNA. In one sample, a *KRAS* mutation was detected in ctDNA, but not in the corresponding tissue. Finally, we tested a third MCN cohort (Figure 1A; Cohort 3, MCN‐3; *n* = 26) with confirmed histopathology, from which only a blood plasma specimen for ctDNA analyses was available. Screening of ctDNA *KRAS* G12/G13 mutations in Cohort 3 revealed 11 of 26 (42.3%) positive cases (Figure 1K), establishing a range of mutant *KRAS* across our liquid‐ and tissue‐based cohorts of ~42%–83% (Figure 1D,I,K).

## Discussion

Our study is the first to provide a thorough characterization of the mutational landscape of as‐yet‐not cancerous, microdissected MCN tissues across several cohorts comprising over 50 cases (tissue, plasma, or both). Specifically, we show: (i) high mutational concordance between MCN and PDAC, with VAF as a putative segregating feature; (ii) no mutational burden could be measured in the neighboring healthy tissue, favoring multifocal clonal events occurring within the same MCN and making an accumulative field cancerization less likely; (iii) the presence of mutant *KRAS* represents a critical oncogenic event in MCN. Notably, the levels of mutant *KRAS*, as reflected by VAF values in microdissected regions, are associated with distinct morphological characteristics that indicate progressive dysplasia; (iv) mutations are heterogeneously distributed across multiple regions of the same MCN, indicating polyclonal events as drivers of intratumor heterogeneity; and (v) a ctDNA‐based liquid biopsy approach can capture, even in low‐grade lesions, oncogenic *KRAS* mutations, albeit to a limited extent.

Genetic characterization of MCN has been attempted, but low ratios between neoplastic cells and dense, cell‐rich stroma can be considered a challenge for the detection of low‐frequency mutations. Consequently, our approach of utilizing targeted sequencing following the microdissection of neoplastic epithelium revealed that 70% of MCNs exhibit somatic alterations in nine PDAC‐related genes, with *KRAS* being the most prominent. This finding aligns with whole‐exome sequencing data from an individual MCN case [2], and also revealed a frameshift mutation in *FBXW7*, along with a novel somatic mutation in *APC* that has not been reported previously [2]. Overall, the lower VAF observed in MCNs suggest a trend towards reduced mutation levels compared to PDAC, implying that the abundance of *KRAS* mutations may represent a key feature of cancer progression. Of note, our WGS (30×) approach of a limited set of MCN did not capture any of the panel sequencing mutations, while only gross structural aberrations (but not single‐gene imbalances) were excluded. In support of this notion, our detailed interrogation of morphologically distinct MCN areas revealed a mosaic of *KRAS*‐mutated regions and nonmutant areas, indicating that the presence of the KRAS mutation *per se* triggers dysplasia. Specifically, our data suggest that either increased mutational dosage in a few cells or the proportion of mutated cells per microdissected area affect typical dysplasia hallmarks like nuclear atypia, columnar cellular architecture, and papillary growth. However, our VAF values might be affected by several factors; for example, the proportion of KRAS‐mutant neoplastic cells versus stromal contamination or mutational dosage gain in individual cells. Our real‐time, pathologist‐supervised microdissection, which positions the laser close to the basement membrane to safeguard the integrity of the neoplastic epithelium and the normalization against the *KRAS* wildtype signal, demonstrate that these values reliably reflect the proportion of mutant cells, even considering potential biases stemming from stromal cellularity. Furthermore, the nearly zero median VAF of mutant *KRAS* observed in the noncolumnar, nonpapillary, and nonpseudo‐stratified epithelial samples (Figure 1J) indicates that the influence of cellularity is likely minimal.

Importantly, the extensive morphologic and genetic heterogeneity in MCN holds promise for liquid biopsy approaches. In our cohort, the ddPCR‐based evaluation of liquid biopsies aiming to assess the MCN *KRAS* genotype returned a sensitivity of just about 50% of lesions, findings that could be further confirmed in an independent liquid biopsy cohort. Still, the ddPCR assay could be deployed as an additional tool for evaluation of dysplasia and the risk of malignant progression in uncertain cystic pancreatic lesions. Our study has further limitations. The panel sequencing approach does not capture the entire coding genome. Besides the few samples analyzed via low‐coverage WGS, DNA of sufficient quality could not be retrieved from the tissue blocks. Also, the predictive value of ddPCR liquid biopsy could be impaired by the presence of mutant *KRAS* originating from other benign lesions, especially PanINs, as shown recently [20], which are known to already harbor oncogenic *KRAS* mutations and were present in the adjacent tissue of our cohort. Additional prospective trials are needed to evaluate the predictive value of mutant *KRAS* liquid biopsy in MCN.

Taken together, our study is one of the largest analyses of human MCN, highlighting: (i) mutational landscapes of tumor epithelium and corresponding adjacent tissue; (ii) intratumor heterogeneity; (iii) *KRAS* mutation abundance as a segregator between invasive cancers and preneoplastic lesions; and (iv) the presence and/or dosage of mutant *KRAS* as a driver of dysplasia.

## Author contributions statement

LAS performed most of the experiments. A Bec performed microdisscection and histopathological analysis as well as imaging. JS and TB assisted A Bec in microdissecting the MCN tissue. RM performed panel sequencing. AWB was involved in study coordination and initial launch of the study. ER made figures and designed schemes. NA and LP drafted and proofread the article. TS provided lab space and proofread the article. N Gi, KK, AB and LP collected clinical data. N Gi coordinated material for Cohorts 1 and 3. N Ga, PM, TB and PS performed the initial histopathological analysis of the PDAC and MCN tissue. SR performed the statistics in the study. TH and MB performed pancreatic surgery and provided samples for Cohorts 1 and 3, while CM performed parts of the pancreatic surgery for Cohort 3. AK designed and supervised the study. LAS, TH and AK drafted the article. All authors proofread the article.

## Supporting information


**Figure S1.** Low coverage whole genome sequencing of an MCN subset detects no copy number variants (CNV) or specific mutations
**Figure S2**. Cohort 2 – MCN #1. Cohort 2, MCN case number 1. Three samples of individual tumor‐containing FFPE tissue blocks were micro‐dissected (A–C) and 7 samples of tumor epithelium and 1 of normal pancreas were analyzed
**Figure S3**. Cohort 2 – MCN #2: Cohort 2, MCN case number 2. Four samples of individual tumor‐containing FFPE tissue blocks were micro‐dissected (A–D) and 7 samples of tumor epithelium and 1 of normal pancreas were analyzed
**Figure S4**. Cohort 2 – MCN #3: Cohort 2, MCN case number 3. Four samples of individual tumor‐containing FFPE tissue blocks were micro‐dissected (A–D) and 6 samples of tumor epithelium and 1 of normal pancreas were analyzed
**Figure S5**. Cohort 2 – MCN #4: Cohort 2, MCN case number 4. Four samples of individual tumor‐containing FFPE tissue blocks were micro‐dissected (A–D) and 16 samples of tumor epithelium and 1 of normal pancreas were analyzed
**Figure S6**. Cohort 2 – MCN #5: Cohort 2, MCN case number 5. Seven samples of individual tumor‐containing FFPE tissue blocks were micro‐dissected (A–G) and 8 samples of tumor epithelium and 1 of normal pancreas were analyzed
**Figure S7**. Cohort 2 – MCN #6: Cohort 2, MCN case number 6. Three samples of individual tumor containing FFPE tissue blocks were micro‐dissected (A–C) and 5 samples of tumor epithelium and 1 of normal pancreas were analyzed
**Figure S8**. Cohort 2 – MCN #7: Cohort 2, MCN case number 7. Seven samples of individual tumor containing FFPE tissue blocks were micro‐dissected (A–G) and 8 samples of tumor epithelium and 1 of normal pancreas were analyzed
**Figure S9**. Cohort 2 – MCN #8: Cohort 2, MCN case number 8. Five samples of individual tumor containing FFPE tissue blocks were micro‐dissected (A–E) and 16 samples of tumor epithelium and 1 of normal pancreas were analyzed
**Figure S10**. Cohort 2 – MCN #9: Cohort 2, MCN case number 9. Four samples of individual tumor containing FFPE tissue blocks were micro‐dissected (A–D) and 5 samples of tumor epithelium and 1 of normal pancreas were analyzed
**Figure S11**. Cohort 2 – MCN #10: Cohort 2, MCN case number 10. Three samples of individual tumor containing FFPE tissue blocks were micro‐dissected (A–C) and 7 samples of tumor epithelium and 1 of normal pancreas were analyzed
**Figure S12**. Cohort 2 – MCN #11: Cohort 2, MCN case number 10. Three samples of individual tumor containing FFPE tissue blocks were micro‐dissected (A–C) and 7 samples of tumor epithelium and 1 of normal pancreas were analyzed
**Figure S13**. Cohort 2 – MCN #12: Cohort 2, MCN case number 12. Five samples of individual tumor containing FFPE tissue blocks were micro‐dissected (A–E) and 14 samples of tumor epithelium and 1 of normal pancreas were analyzed.

**Table S1.** Demographic data of Cohorts 1–3

**Table S2.** Histopathological details of Cohorts 1–3

**Table S3.** Tabular summary of the *KRAS* mutation VAF values across all 114 micro dissected MCN regions according to histomorphological features and intratumor heterogeneity

## Data Availability

There are restrictions to the availability of the whole‐genome sequencing data, which cannot be publicly shared due to their germ line traceability. The raw sequencing data from the four MCN samples is securely stored at the EMBL Heidelberg and will be shared by the lead contact (Prof. Dr. Alexander Kleger) and the operating scientists (Dr. Tobias Rausch, Dr. Vladimir Benes) upon request.
