# Supplementary material for: Unveiling the intriguing relationship: oncogenic KRAS , morphological shifts, and mutational complexity in pancreatic mucinous cystic neoplasms
Source: J Pathol. 2025 Feb 5;265(4):401–7. doi: 10.1002/path.6397 (PMC11880986; doi:10.1002/path.6397)
Supplement: Supplementary file 1 — Figure S1. Low coverage whole genome sequencing of an MCN subset detects no copy number variants (CNV) or specific mutations Figure S2. Cohort 2 – MCN #1. Cohort 2, MCN case number 1. Three samples of individual tumor‐containing FFPE tissue blocks were micro‐dissected (A–C) and 7 samples of tumor epithelium and 1 of normal pancreas were analyzed Figure S3. Cohort 2 – MCN #2: Cohort 2, MCN case number 2. Four samples of individual tumor‐containing FFPE tissue blocks were micro‐dissected (A–D) and 7 samples of tumor epithelium and 1 of normal pancreas were analyzed Figure S4. Cohort 2 – MCN #3: Cohort 2, MCN case number 3. Four samples of individual tumor‐containing FFPE tissue blocks were micro‐dissected (A–D) and 6 samples of tumor epithelium and 1 of normal pancreas were analyzed Figure S5. Cohort 2 – MCN #4: Cohort 2, MCN case number 4. Four samples of individual tumor‐containing FFPE tissue blocks were micro‐dissected (A–D) and 16 samples of tumor epithelium and 1 of normal pancreas were analyzed Figure S6. Cohort 2 – MCN #5: Cohort 2, MCN case number 5. Seven samples of individual tumor‐containing FFPE tissue blocks were micro‐dissected (A–G) and 8 samples of tumor epithelium and 1 of normal pancreas were analyzed Figure S7. Cohort 2 – MCN #6: Cohort 2, MCN case number 6. Three samples of individual tumor containing FFPE tissue blocks were micro‐dissected (A–C) and 5 samples of tumor epithelium and 1 of normal pancreas were analyzed Figure S8. Cohort 2 – MCN #7: Cohort 2, MCN case number 7. Seven samples of individual tumor containing FFPE tissue blocks were micro‐dissected (A–G) and 8 samples of tumor epithelium and 1 of normal pancreas were analyzed Figure S9. Cohort 2 – MCN #8: Cohort 2, MCN case number 8. Five samples of individual tumor containing FFPE tissue blocks were micro‐dissected (A–E) and 16 samples of tumor epithelium and 1 of normal pancreas were analyzed Figure S10. Cohort 2 – MCN #9: Cohort 2, MCN case number 9. Four samples of individual tumor [file PATH-265-401-s002.docx]

**Unveiling the intriguing relationship: oncogenic *KRAS*, morphological shifts, and mutational complexity in pancreatic mucinous cystic neoplasms**

LA Schulte *et al. J Pathol* <https://doi.org/10.1002/path.6397>

**Supplementary Figures S1–S12**

**Supplementary Tables S1–S3 are provided as separate Excel files**

**A**

**Figure S1.** Low-coverage whole-genome sequencing of an MCN subset detects no copy number variants (CNV) or specific mutations.

**Figure S2. Cohort 2 – MCN #1:** Cohort 2, MCN case number 1. Three samples of individual tumors containing FFPE tissue blocks were microdissected (A–C) and seven samples of tumor epithelium and one of normal pancreas were analyzed. Histologic evaluation showed areas with cuboidal and columnar morphology and basal nuclei; see supplementary material, Table S2.

**Figure S3. – Cohort 2 – MCN #2:** Cohort 2, MCN case number 2. Four samples of individual tumor-containing FFPE tissue blocks were microdissected (A–D) and seven samples of tumor epithelium and 1 of normal pancreas were analyzed. Histologic evaluation showed areas with cuboidal and columnar morphology and basal nuclei as well as focal pseudo-stratification; see supplementary material, Table S2.

**Figure S4. – Cohort 2 – MCN #3:** Cohort 2, MCN case number 3. Four samples of individual tumor-containing FFPE tissue blocks were microdissected (A–D) and six samples of tumor epithelium and 1 of normal pancreas were analyzed. Histologic evaluation showed areas with cuboidal and columnar morphology and basal nuclei cuboidal and columnar morphology and basal nuclei; see supplementary material, Table S2.

**Figure S5. – Cohort 2 – MCN #4:** Cohort 2, MCN case number 4. Four samples of individual tumor-containing FFPE tissue blocks were microdissected (A–D) and 16 samples of tumor epithelium and one of normal pancreas were analyzed. Histologic evaluation showed areas with cuboidal and columnar morphology and focal papillary growth and pseudo-stratified nuclei; see supplementary material, Table S2.

**Figure S6. – Cohort 2 – MCN #5:** Cohort 2, MCN case number 5. Seven samples of individual tumor-containing FFPE tissue blocks were microdissected (A–G) and eight samples of tumor epithelium and one of normal pancreas were analyzed. Histologic evaluation showed areas with cuboidal and columnar morphology and focal papillary growth and pseudo-stratified nuclei; see supplementary material, Table S2.

**Figure S7. – Cohort 2 – MCN #6:** Cohort 2, MCN case number 6. Three samples of individual tumor containing FFPE tissue blocks were microdissected (A–C) and five samples of tumor epithelium and one of normal pancreas were analyzed. Histologic evaluation showed areas with cuboidal and columnar morphology and basal nuclei; see supplementary material, Table S2.

**Figure S8. – Cohort 2 – MCN #7:** Cohort 2, MCN case number 7. Seven samples of individual tumor containing FFPE tissue blocks were microdissected (A–G) and eight samples of tumor epithelium and one of normal pancreas were analyzed. Histologic evaluation showed areas with cuboidal and columnar morphology and basal nuclei; see supplementary material, Table S2.

**Figure S9. – Cohort 2 – MCN #8:** Cohort 2, MCN case number 8. Five samples of individual tumor containing FFPE tissue blocks were microdissected (A–E) and 16 samples of tumor epithelium and one of normal pancreas were analyzed. Histologic evaluation showed areas with cuboidal and columnar morphology and focal papillary growth and pseudo-stratified nuclei; see supplementary material, Table S2.

**Figure S10. – Cohort 2 – MCN #9:** Cohort 2, MCN case number 9. Four samples of individual tumor containing FFPE tissue blocks were microdissected (A–D) and five samples of tumor epithelium and one of normal pancreas were analyzed. Histologic evaluation showed areas with cuboidal and columnar morphology and basal nuclei; see supplementary material, Table S2.

**Figure S11. – Cohort 2 – MCN #10:** Cohort 2, MCN case number 10. Three samples of individual tumor containing FFPE tissue blocks were microdissected (A–C) and seven samples of tumor epithelium and one of normal pancreas were analyzed. Histologic evaluation showed areas with cuboidal and columnar morphology and focal papillary growth as well as associated cancer; see supplementary material, Table S2.

**Figure S12. – Cohort 2 – MCN #11:** Cohort 2, MCN case number 10. Three samples of individual tumor containing FFPE tissue blocks were microdissected (A–C) and seven samples of tumor epithelium and one of normal pancreas were analyzed. Histologic evaluation showed areas with cuboidal and columnar morphology and focal papillary growth as well as associated cancer; see supplementary material, Table S2.

**Figure S13. – Cohort 2 – MCN #12:** Cohort 2, MCN case number 12. Five samples of individual tumor containing FFPE tissue blocks were microdissected (A–E) and 14 samples of tumor epithelium and one of normal pancreas were analyzed. Histologic evaluation showed areas with cuboidal and columnar morphology and focal papillary growth and pseudo-stratified nuclei; see supplementary material, Table S2.
